# Supplementary material for: Chromothripsis during telomere crisis is independent of NHEJ, and consistent with a replicative origin
Source: Genome Res. 2019 May;29(5):737–49. doi: 10.1101/gr.240705.118 (PMC6499312; doi:10.1101/gr.240705.118)
Supplement: Supplemental Material [file supp_gr.240705.118_Supplemental_file_1.zip › contigs/annotated_contigs/DB95/contig.2.DB95_length_469_mean_cov_8.96801705757.docx]

**DB95_length_469_mean_cov_8.96801705757**

```
CCTCCCCGTTTTTTTGTTTTTTTTTTTTTGAGAAGTAGTCTCACTCTGTTGCACAGGCTGGAGTGCAGTGGCATGATCTCCGCTCACTG
 >chr17:40777731-40778032 + E=2e-133
CAAGCTCCGCCTCCCGGGTTCAGGCCATTCTCCTGCCTCAGCCTCCCGAGTAGCTGGGACTACAGGCGCCCGCCACCACGCCCGGCTAA

TTTTTTGTATTTTTAGTAGAGACAGGGTTTCACCATGTTAGCCAGGATGGTCTCGATCTCCTGACCTCGTGATCCGCCCGCCTCGGCCT

CCCAAAGTGCTGGGATTACAGGCGTGAGCCACCA|TGCCCTGCCAGAATGAGCACCTCTTCACAGGGAGTACACAAGCTCTGAGGAGAT

CGGAAGAGCGTCGTGTAGGGAAAGAGTGTTCAGAGCCGTGTAGATCTCGGTGGTCGCCGTATCATTAAAAA|ACAGCGTGGGTAACAAG
                                                                        >chr5:131065451-
AGCGAAGCTCTGTCTCAAAA|TATATA131065488 - E=1e-04
```
